# Autoantibody-mediated pain in long COVID: evidence from multiple lines

**DOI:** 10.3389/fimmu.2026.1840110

**Published:** 2026-06-17

**Authors:** Charles Nicaise, Pierre Bulpa, Marc Jamoulle

**Affiliations:** 1 URPhyM, NARILIS, Université de Namur, Namur, Belgium; 2 Intensive Care Unit, CHU UCL Namur, Yvoir, Belgium; 3 HEC Information Sciences, Université de Liège, Belgium, Liège, Belgium

**Keywords:** allodynia and hyperalgesia, autoantibody, long Covid, pain, post-acute sequela of COVID-19 (PASC), preclinical (*in vivo*) studies

Early during the SARS-CoV-2 pandemic, it became evident that a subset of infected individuals developed persistent, long-term sequelae, now collectively referred to post-acute sequelae of COVID-19 or long COVID, a multifactorial condition whose definition is constantly evolving, according to WHO and NASEM (1). To date, part of the medical community still doubts about the existence of the disease, or at least about underlying biological mechanisms, and remains largely unequipped to provide comprehensive or personalized care for these patients.

Long COVID is increasingly recognized as a clinically heterogeneous condition encompassing a wide spectrum of persistent symptoms, including fatigue, dyspnea, cognitive impairment, autonomic dysfunction, and chronic pain, with substantial variability in symptom clusters, severity, and duration across patients. Emerging evidence also highlights marked sex differences, as women appear disproportionately affected by long COVID and are more likely to develop multisystem and pain-related phenotypes, potentially reflecting sex-dependent differences in immune responses, hormonal regulation, and susceptibility to autoimmune or central sensitization processes. Despite the growing prevalence and clinical burden, its underlying pathomechanisms remain incompletely understood. Current hypotheses implicate a complex interplay between persistent immune activation, viral persistence, neuroinflammation, endothelial dysfunction, dysautonomia, and autoimmunity, but no unifying mechanism has yet been established. This incomplete mechanistic understanding continues to limit the development of reliable biomarkers and phenotype-specific diagnostics. Distinct immune and autoimmune profiles in patients have recently been reported, further supporting the existence of different long COVID endotypes (2, 3).

In the aftermath of the first lockdowns, the global re-synchronization of research efforts has led, independently across four research groups (UMC Utrecht, NL; Yale University, USA; Université de Namur, BE; King’s College London, UK), to the hypothesis that a proportion of long COVID symptoms may arise from autoimmune mechanisms mediated by pathogenic autoantibodies. In this context, recent publications and preprints investigated the link between immunoglobulin G (IgG) derived from patients and sensory deficits or pain-related phenotypes (2–5). These studies set up comparable experimental paradigms, including passive transfer of human immunoglobulins into laboratory mice, behavioral phenotyping, identification of target tissues and antigenic binding sites, and characterization of the autoantibody repertoire. The findings, despite minor differences in experimental design (i.e. human IgG doses), converged to show that patient IgG triggered mechanical allodynia and thermal hyperalgesia in transferred mice (2–5). Within a short time frame—ranging from weeks to months— the preliminary data were disseminated as abstracts or preprints via open-access platforms, thereby fostering rapid scientific exchange and stimulating research efforts. Importantly, the robustness and impact of these findings are strengthened by their replication across geographically distinct labs and on independent patient cohorts.

This apparent coincidence of timing reflects a well-established concept in the sociology of science known as “multiples in scientific discovery”, described by Robert K. Merton (6). According to this framework, independent and simultaneous discoveries become likely when a shared intellectual climate and enabling conditions are gathered. Historical examples include the independent development of infinitesimal calculus by, one side, Isaac Newton and, another side, Gottfried Wilhelm Leibniz, as well as the description of a theory of evolution by Charles Darwin and in parallel by Alfred Russel Wallace. In contrast to earlier centuries, modern tools for rapid data sharing (e.g., bioRxiv, medRxiv, Research Square) and global networking have dramatically increased the likelihood of such synchronous discoveries. Beyond fostering healthy competition, such independent yet convergent research ultimately strengthens scientific evidence through rigor and reproducibility. These current advances also challenge interpretations that do not fully account for biological mechanisms in long COVID (7).

Overall, the converging findings provide strong support for a biological basis underlying post-acute COVID-19 multisystem dysfunction. Although multiple mechanisms may coexist, an antibody-mediated autoimmune process—triggered during the acute phase of SARS-CoV-2 infection—may account for peripheral nervous system disturbances, such as those observed in autoimmune sensory neuropathies or neuronopathies. While caution is still warranted given the preclinical nature of these studies, the lack of identification of a causal or universal autoantibody still jeopardizes the translation into a single therapy. The autoantibody repertoire is quite heterogeneous across patients included in the studies (2–4), but binding and functional IgG assays in the animal model point towards the involvement of dorsal root ganglion sensory neurons (4) or their distal afferences, some of which extend to intraepidermal nerve fibers (3, 5). These observations deserve further investigation using skin biopsy samples or electrodiagnostic testing in informed patients, as well as validation on large cohorts. These mechanistic insights nevertheless open therapeutic avenues aimed at neutralizing pathogenic antibodies, eliminating autoreactive cells, or dampening persistent immune activation (e.g. plasmapheresis, intravenous immunoglobulin (IVIg) therapy, neonatal Fc receptor blockade, immunoadsorption, anti-CD20–mediated B-cell depletion, immunosuppressive drugs). In this context, the development of biomarkers capable of stratifying patients with autoimmune-mediated pain endotypes — for instance through the identification of pathogenic autoantibodies — would be instrumental in refining patient selection and personalizing therapeutic interventions. To improve comparability and reproducibility across both preclinical and clinical trials, it is essential to comprehensively report patient demographics (vaccination status, SARS-CoV-2 variants, co-morbidities, …), inclusion/exclusion criteria, description of relevant symptoms using Human Phenotype Ontology classifiers, and diagnostic definitions, as variations in cohort selection and case characterization can substantially influence data interpretation and study outcomes. Although exploratory studies have showcased beneficial effects of IVIg in small cohorts (8), such approaches are unlikely to be universally effective across the heterogeneous spectrum of the disease. So far, no evidence-based guidelines exist to help clinicians identify which patients are most likely to benefit from immunomodulatory therapies, nor are there reliable predictors of therapeutic responsiveness.
